# Imaging in Vascular Liver Diseases

**DOI:** 10.3390/medicina60121955

**Published:** 2024-11-27

**Authors:** Matteo Rosselli, Alina Popescu, Felix Bende, Antonella Al Refaie, Adrian Lim

**Affiliations:** 1 Department of Internal Medicine, San Giuseppe Hospital, USL Toscana Centro, 50053 Empoli, Italy; antonellafardus.alrefaie@uslcentro.toscana.it; 2 Division of Medicine, Institute for Liver and Digestive Health, University College London, London NW3 2PF, UK; 3 Department of Gastroenterology and Hepatology, “Victor Babeș” University of Medicine and Pharmacy Timișoara, Eftimie Murgu Square 2, 300041 Timisoara, Romania; 4 Department of Medicine, Surgery and Neuroscience, University of Siena, 53100 Siena, Italy; 5 Imperial College London and Healthcare NHS Trust, London SW 2AZ, UK

**Keywords:** vascular liver diseases, portal vein thrombosis, outflow obstruction syndrome, porto-sinusoidal vascular disorder, vascular malformations, Osler–Weber–Rendu disease

## Abstract

Vascular liver diseases (VLDs) include different pathological conditions that affect the liver vasculature at the level of the portal venous system, hepatic artery, or venous outflow system. Although serological investigations and sometimes histology might be required to clarify the underlying diagnosis, imaging has a crucial role in highlighting liver inflow or outflow obstructions and their potential causes. Cross-sectional imaging provides a panoramic view of liver vascular anatomy and parenchymal patterns of enhancement, making it extremely useful for the diagnosis and follow-up of VLDs. Nevertheless, multiparametric ultrasound analysis provides information useful for differentiating acute from chronic portal vein thrombosis, distinguishing neoplastic invasion of the portal vein from bland thrombus, and clarifying the causes of venous outflow obstruction. Color Doppler analysis measures blood flow velocity and direction, which are very important in the assessment of VLDs. Finally, liver and spleen elastography complete the assessment by providing intrahepatic and intrasplenic stiffness measurements, offering further diagnostic information.

## 1. Introduction

VLDs are a heterogeneous group of disorders affecting the liver vasculature, leading to different clinical scenarios depending on the vessel(s) involved, the site and extension of involvement, and the onset of development (i.e., acute, subacute, chronic). VLDs can affect the portal venous system, hepatic artery, or impair hepatic venous outflow at the level of the sinusoids, small hepatic venules, hepatic veins, or the inferior vena cava. Under certain circumstances, and depending on the underlying disorder, a combination of involved vessels can also be observed. Portal hypertension (PH) often develops as a consequence of increased resistance/blockage of hepatic blood flow, or a significant increase in hepatic arterial flow from vascular malformations (VMs), such as arterial-portal or arterial-venous shunting. Imaging findings of PH or VMs in different VLDs are an expression of the site and vessels involved and provide important clues about the etiology of the disease. Liver parenchyma can undergo profound modifications due to perfusion and fibrotic changes, contrasting with cirrhosis, where vascular involvement and PH are never the primary cause but a consequence of fibrosis and nodular regeneration. Depending on the type of VLD, liver function can also be impaired, with its severity usually correlating with the site, extension, and especially the onset of vascular involvement. This review provides information on the importance of the use of different imaging modalities in the diagnosis of VLDs, particularly emphasizing ultrasound and its multiparametric assessment, as well as the benefit of integrating different imaging modalities.

## 2. Portal Vein Thrombosis

Portal vein thrombosis (PVT) is defined by a partial or complete thrombotic occlusion of the portal venous system. It is classified according to the site, extension and onset of the thrombosis. Regarding extension, it is important to describe whether this is confined to one branch or involves two branches, or the extrahepatic trunk, as well as whether the splenic and/or mesenteric veins are involved [1,2]. The diagnostic workup should take into consideration the pathophysiological background related to the presence or absence of cirrhosis and exclude neoplastic invasion of the vessel and the presence of a thrombophilic state, which can depend on constitutional, inherited, or acquired factors, as well as superimposed causes triggering the coagulation cascade [3].

Imaging plays a crucial role in the diagnosis and classification of PVT for which contrast-enhanced computer tomography (CECT) is an excellent diagnostic method. It provides a panoramic view of the whole abdomen and splanchnic circulation and is important not only for classification purposes but also for highlighting sites of ischemia and underlying diseases. It is also extremely useful for the planning of interventional strategies that may be required in selected cases, as well as for providing information on the technical feasibility of liver transplantation in case of extrahepatic involvement of the portal venous system. In addition, CECT negates the presence of any acoustic barriers typically caused by air in the gastrointestinal tract, which represents a significant limitation for sonographic assessment. The downsides of CECT are radiation exposure and its contraindications in patients with renal failure. While radiation exposure does not represent a contraindication for contrast-enhanced MRI (CEMRI), renal failure was a concern for the potential development of nephrogenic systemic sclerosis until the development of macrocyclic agents [4]. However, CEMRI is also more costly, and its acquisition time is slower [1]. Regardless of the clear benefits of cross-sectional imaging providing a structural and topographical overview of PVT, ultrasound imaging with color Doppler has an excellent accuracy and has been promoted as the first-line imaging modality to diagnose and follow-up PVT [5]. It is highly accurate in assessing the texture of thrombus, which correlates with the onset of its formation, as well as the presence of cavernous transformation, residual flow and its direction, and the presence of VMs, which can develop as a consequence of severe PH. The adjunct of contrast-enhanced ultrasound (CEUS) is extremely accurate in differentiating bland thrombus from the presence of neoplastic tissue invading the portal vein, similarly to CECT and CEMRI [6]. Moreover, CEUS is a tool to be used when other imaging modalities are contraindicated, or as a complementary investigation to inconclusive CECT and CEMRI studies. Radiation exposure is also reduced when close and frequent follow-up is required.

**Figure 1:**
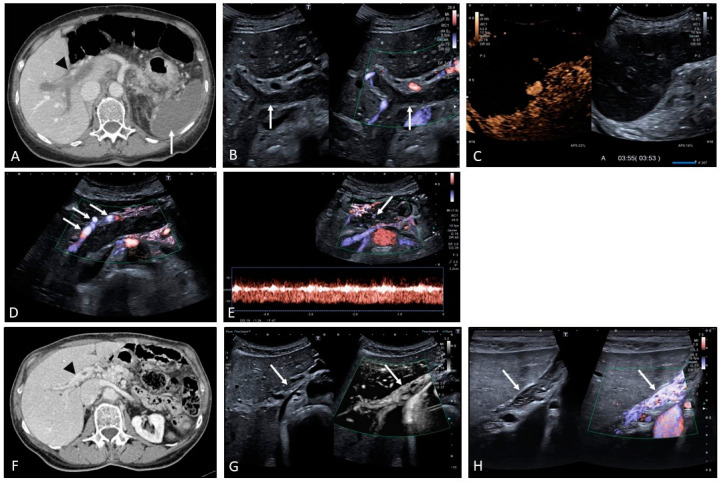
Acute splanchnic vein thrombosis with extensive involvement of the mesenteric, splenic, and portal venous system. The image provided in (**A**) shows a transverse section of the liver and spleen on contrast-enhanced CT (CECT) showing thrombosis of the portal venous system (hypodense material filling the vascular lumen, arrowhead). Note is made of complete un-enhancement of the spleen in keeping with subtotal splenic ischemic infarction (arrow). B-mode ultrasound images integrated by directional power doppler show the clot corresponding to hypoechoic material that fills the portal vein, including its intrahepatic bifurcation ((**B**), arrows). Contrast-enhanced ultrasound reveals a ‘black spleen’ (**C**) corresponding to the complete absence of intrasplenic residual vascularity seen on CT (**A**). The patient was immediately commenced on anticoagulation treatment and followed up with sequential imaging. After 2 weeks there is evidence of increased arterial hypertrophy around the clot ((**D**), arrows) and initial signs of cavernous recanalization as revealed by the evidence of a portal venous flow trace within the clot ((**E**), arrow). (**F**) A CECT at 12 months distance revealed cavernous transformation of the portal vein (arrowhead) with good flow. Microvascular imaging and directional power doppler show the portal flow running through a thin fibrin reticulate as a result of the re-canalized thrombus ((**G**,**H**), arrows).

**Figure 2:**
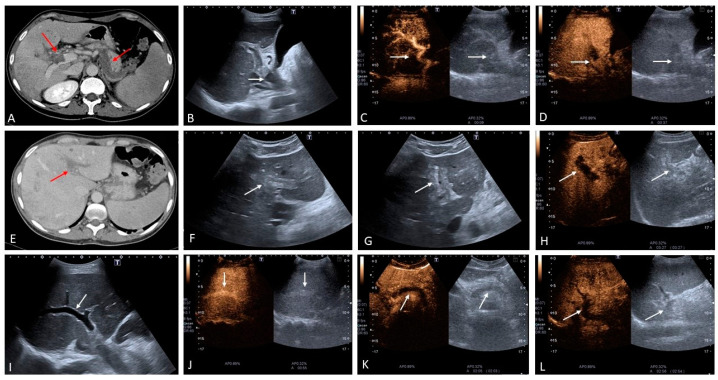
Acute portal vein thrombosis in a patient with polycythemia rubra vera. (**A**) Contrast-enhanced CT scan shows a clear sign of partial thrombosis of the extrahepatic portal venous trunk, complete thrombosis of the right anterior portal branch and splenic vein (red arrows). On B-mode ultrasound a clear demarcation of the site of thrombosis can be observed ((**B**), white arrow). Contrast-enhanced ultrasound (CEUS) shows pronounced hypertrophy of the hepatic artery with arterial buffering revealed by its hyperenhancement on the background of portal hypoperfusion ((**C**), white arrows), with evidence of thrombosis of the right anterior portal branch ((**D**), the white arrows highlights the boundary between the thrombosed and patent portal vein). The left portal vein branch is completely thrombosed as shown on CECT ((**E**) red arrow), B-mode ultrasound ((**F**,**G**), white arrows) and CEUS ((**H**), white arrow). Patency of the right posterior branch of the portal vein is also confirmed on B-mode ((**I**), white arrow) and CEUS ((**I**,**J**), white arrows). There is complete thrombosis of the splenic vein with consequent splenic hypoperfusion ((**K**,**L**), white arrows).

**Figure 3:**
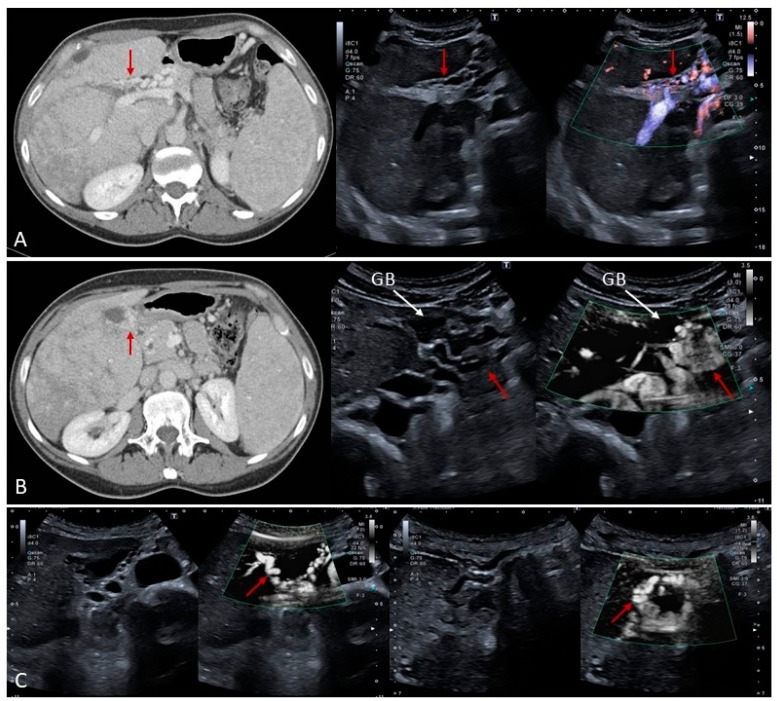
Months after acute portal vein thrombosis (showed in Figure 2), there is evidence of cavernous transformation of the anterior branch of the right portal vein as it can be seen in both the contrast-enhanced CT scan and directional power Doppler ((**A**) red arrows, left and right side of the figure, respectively). Pericholecystic varices have also developed ((**B**), white arrows point to the gallbladder (GB); red arrows point to the pericholecystic varices). Ultrasound microvascular imaging highlights the details of the varicosities ((**C**), red arrows).

Acute PVT can be secondary to many causes, ranging from acquired to inherited causes of thrombophilia [5]. Depending on its onset and extension, it can present clinically with an acute abdomen as a cause of ischemic injury that may be especially life-threatening in cases where extensive thrombosis occludes the mesenteric veins, thus causing bowel ischemia. In other circumstances, it can be more subtle and almost asymptomatic, presenting incidentally on routine ultrasound or cross-sectional imaging carried out for other reasons. Alternatively, chronic PVT can cause subclinical development of non-cirrhotic portal hypertension and then present initially with complications such as variceal bleeding and, less commonly, ascites. In the acute phase, CECT identifies the thrombus as hypodense material filling the lumen of the portal vein, with downstream arterial hyperemia of the parenchymal segment that is not receiving portal venous perfusion (‘deportalization’ of liver parenchyma). Prompt detection in the acute phase is crucial, facilitating early recanalization of the clot, or at least part of it, if anticoagulation is started immediately. At the same time, it makes it possible to select patients who would benefit from interventional management, which in some cases may prove to be lifesaving, thus significantly affecting prognosis. Ultrasound can easily detect the portal venous clot which, in the acute phase, is usually hypoechoic, mostly homogeneous, with no intra-thrombotic signs of vascularization, and tends to be surrounded by intense compensatory arterial hypertrophy. Eventually, if the clot is not completely occlusive, blood flow can be detected around it. With time, the echogenicity of the thrombus changes, becoming more pronounced and echogenic. The development of a portal vein cavernoma occurs quite rapidly (usually within 2–3 weeks) as the consequence of acute occlusion, when the portal flow manages to create vascular channels that develop around and, in some cases, within the clot when it becomes recanalized. In other circumstances, PVT may leave an intrahepatic fibrotic remnant, with pronounced arterial buffering and severe pre-hepatic PH with upstream portal systemic shunting (Figure 1 and Figure 2). In case of extrahepatic portal vein obstruction, the cystic vein that drains into the portal venous system increases in size and the gallbladder becomes congested and thickened. Often pericholecystic varices, as well as portal systemic shunts, develop at this level (Figure 3) [7].

**Figure 4:**
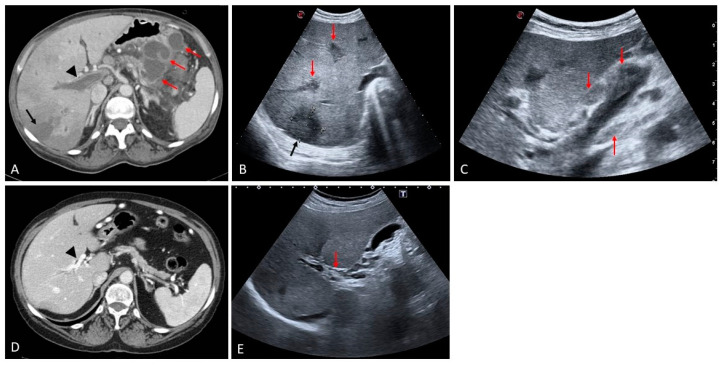
Subacute pancreatitis complicated by infected pseudocysts and portal vein thrombophlebitis (pylephlebitis). (**A**) On contrast-enhanced CT (CECT) the red arrows highlight the infected pseudocysts. Note is made of enhancement of the portal vein walls (arrowhead) and segment VII large hypoperfusional area (black arrow) in the context of which a hypoechoic rounded collection (calipers) is well identified on ultrasound ((**B**), black arrow). Distal anterior and posterior thrombosed portal venous branches ((**B**), red arrows). Hypoechoic thrombus is filling the main portal vein with extensive thickening of its walls (**C**). Multiple reactive lymphadenopathies are also present (red arrows). At one-year from onset portal vein cavernous transformation is seen on both (CECT) ((**D**), arrowhead) and B-mode ultrasound ((**E**), red arrow).

**Figure 5:**
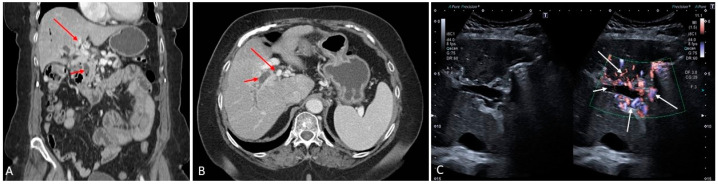
Patient with abdominal discomfort and a biochemical picture of cholestasis with a previous history of pylephlebitis. Contrast-enhanced CT showed pronounced varicosities compatible with multiple convoluted vascular channels as a result of longstanding portal vein thrombosis with cavernous transformation surrounding dilated bile ducts compatible with portal biliopathy ((**A**,**B**) long red arrows). The thrombosed portal vein cannot be visualized and is likely to have undergone fibrotic retraction. The hypodense channel represents the dilated common bile duct ((**A**,**B**), short red arrows). The ultrasound images (**C**) highlight the dilated CBD (short white arrow) surrounded by numerous collaterals from the cavernous transformation (long red arrows).

A distinct cause of acute PVT is pylephlebitis which is a septic thrombophlebitis of the portal vein. The most common cause of this condition is a diverticular abscess, but it can also be caused by appendicitis, cholangitis, pancreatitis, or suppurative peritonitis [8]. One of its main distinguishing features is PVT but with thickening and enhancement of the portal vein walls, as well as periportal oedema. CECT also usually allows for the evaluation of the extension of the thrombosis and for the identification of the infective source, as well as the presence of the thrombotic extension within the mesenteric vein and possible signs of bowel ischemia. Sonography is also extremely accurate and permits visualization of any portal vein wall thickening and oedema, arterial buffering, and in many instances, reactive lymphadenopathy, which can be seen at the level of the hepatic hilum and along the hepatoduodenal ligament (Figure 4). The development of a portal vein cavernoma occurs in the chronic stages of acute PVT in a non-cirrhotic liver, regardless of the underlying cause, and can lead to portal biliopathy (Figure 5) [9]. In fact, the presence of a large portal vein cavernoma can cause indentation of the bile ducts, leading to wall irregularity, smooth strictures with upstream dilatation, and luminal filling defects. Bile duct strictures with upstream dilatation may also be the result of compression by the dilated tortuous collaterals [7]. Portal biliopathy can be asymptomatic, with mild elevation of cholestatic markers, or manifest with obstructive jaundice and cholangitis. Management of symptomatic cases entails cautious biliary stenting, as well as interventional procedures to reduce portal pressure, such as surgical or transjugular portosystemic shunting. As mentioned, and as shown in Figure 3, pericholecystic varices are a common finding in portal vein obstruction, secondary to PVT and cavernous transformation [7].

Neoplastic invasion of the portal vein is often known as ‘neoplastic portal vein thrombosis’. Nevertheless, in this case, the portal vein is not occluded by thrombotic material but by neoplastic tissue. The most frequent cause is hepatocellular carcinoma on the background of a cirrhotic liver. However, it can also be caused by cholangiocarcinoma, metastatic gastrointestinal disease, pancreatic adenocarcinoma, and neuroendocrine tumors. The features pointing to neoplastic invasion of the portal vein are the following:

(i) the presence of primary or secondary liver malignancy and echogenic material filling the portal venous lumen;
(ii) the diameter of the lumen of the portal vein is often increased according to the growth of the tumor within the vessel;
(iii) evidence of vascularization of the thrombus as a consequence of neoplastic neoangiogenesis [1].

**Figure 6:**
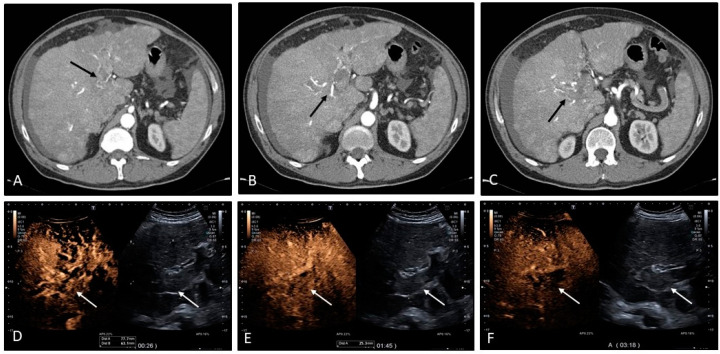
Patient with advanced cirrhosis and diffuse nodularities that enhance in the arterial phase. Of note are the presence of extensive intrahepatic portal vein thromboses that show signs of arterialization on contrast-enhanced CT scan ((**A**–**C**), black arrows). Contrast-enhanced ultrasound shows rapid contrast enhancing of the thrombosed portal vein and subsequent washout in the portal and late vascular phase ((**D**–**F**), white arrows). The findings of enhancement and washout are compatible with neoplastic invasion of the portal vein.

**Figure 7:**
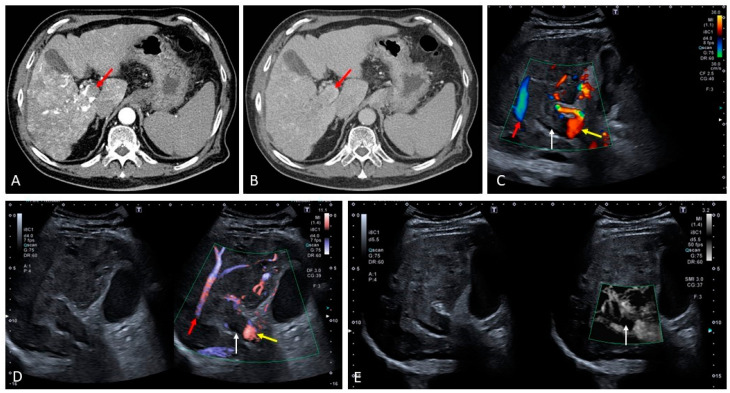
Portal vein thrombosis of the right portal venous branch in a cirrhotic patient and a large downstream heterogeneously perfused area characterized by multiple large pseudonodularities and pronounced arterial buffering. Note is made of intra-thrombotic arterial branching on contrast-enhanced CT and the large dysperfusional area within the right lobe. (**A**), corresponds to the arterial phase of contrast-enhanced CT and (**B**), the venous phase. The red arrows point to the right portal venous thrombus. In (**C**,**D**), colour and directional power Doppler highlight the presence of the thrombus (white arrows) and the upstream flow before the thrombus (yellow arrow). Note is made of the right hepatic vein (red arrow) that crosses the area without being significantly distorted. If there was neoplastic growth, the hepatic vein would have probably been invaded or displaced, which is not seen in this case. Microvascular imaging highlights microscopic vascularity within the thrombus, making it suspicious for neoplasia ((**E**) white arrow).

**Figure 8:**
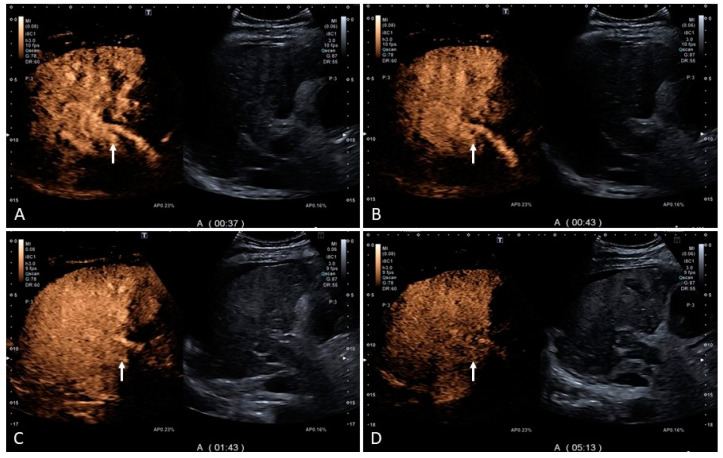
Contrast-enhanced ultrasound (CEUS) of the same case presented in Figure 7. An ‘arterialized’ thrombus should always raise the suspicion of ‘neoplastic vascular invasion’. Contrast-enhanced imaging is usually very accurate at showing arterial enhancement with washout in the portal and subsequent late vascular phases in case of neoplastic invasion. However, one of the pitfalls on CEUS is that intra-thrombotic arterialization as a mechanism of pronounced buffering can mimic arterial enhancement of neoplastic tissue invading the portal vein. In fact, no sign of washout is seen in the portal and late vascular phase in this case ((**A**–**D**), white arrows). There was no evidence of neoplasia.

**Figure 9:**
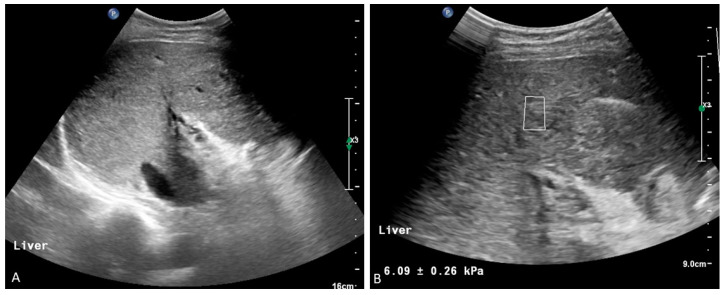
Longstanding portal vein thrombosis has caused considerable heterogeneity of the liver parenchyma (**A**,**B**). Liver stiffness measured by point wave shear wave elastography shows a normal value, ruling out significant fibrosis (**B**).

Irrespective of these findings, the presence of contrast enhancement of the ‘thrombus’ with subsequent washout in the portal and late vascular phase is pathognomonic of neoplastic portal vein invasion [6,10,11,12]. CEUS is extremely accurate and has been shown to be comparable to both CECT and CEMRI—and in some cases even better—by providing real-time evaluation of the enhancement of the different vascular phases (Figure 6). However, there are some cases that are more challenging to interpret. These are usually found in cirrhosis owing to the vascular anomalies that can develop in severe PH complicated by PVT. More specifically, PVT in cirrhosis occurs on the background of a significant reduction of portal flow velocity. It has been shown that a cutoff of portal flow velocity <15 cm/s is associated with an increased risk of PVT [13,14,15]. In cases where PVT develops, and depending on the onset and reorganization of the vascular inflow, a markedly hypertrophic hepatic artery with pronounced branching at the level of the liver hilum and thrombosed portal vein can occur to compensate for the reduced/absent portal venous inflow (Figure 7). From a diagnostic point of view, this appearance can be misleading and confused with portal vein cavernous transformation, which instead is mainly secondary to an intricate system of portal vascular channels that bypass the thrombosed portal vein. Alternatively, it may also give a ‘false’ positive diagnosis of neoplastic thrombus due to the signal of the arterial hyperenhancement of the hypertrophic hepatic artery around—and sometimes within—the thrombus, which can be mistaken for enhancement of the thrombus itself. However, while the presence of compensatory arterial flow may mimic PVT enhancement, there will not be any sign of washout in this case, which is a distinctive hallmark of ‘tumor within a thrombus’ (Figure 8 and Figure 9).

In some cases, it may be difficult to distinguish the morphological appearance of a liver with longstanding PVT from a cirrhotic liver. In fact, chronic PVT can lead to parenchymal changes and a morphological appearance that can resemble cirrhosis. Often the parenchyma may have a heterogeneous echotexture and be significantly arterialized; there may also be caudate lobe hypertrophy and mild irregularity of the liver outline. In these cases, elastography is useful, since liver stiffness will be relatively lower in patients with longstanding PVT that occur in a non-cirrhotic liver, despite the pseudo-cirrhotic appearance (Figure 9) [16].

In conclusion, PVT can be acute, chronic, or it can be mimicked by a tumor invading the portal vein. It can occur in a healthy liver or in cirrhosis. Imaging can evaluate its extent, monitor complications, or assess response to treatment. It may delineate features that can be related to a specific cause. Elastography assessment of liver parenchyma enables the exclusion of significant chronic liver disease.

## 3. Porto-Sinusoidal Vascular Disorder

Porto-sinusoidal vascular disorder (PSVD) encompasses a group of vascular liver diseases characterized by histological abnormalities that involve the portal venules and sinusoids. It is associated with autoimmune, toxic, infective, and inflammatory conditions, as well as recently described genetic predisposition [17,18]. Although PH may not yet have developed at the time of diagnosis, it is a common feature in the majority of cases, and patients are often found with splenomegaly and thrombocytopenia, or specific findings of PH, such as portosystemic vascular collaterals. Under other circumstances, PSVD may manifest with an acute event related to severe PH, such as variceal bleeding or ascites, while portal-systemic hepatic encephalopathy may rarely occur.

**Figure 10:**
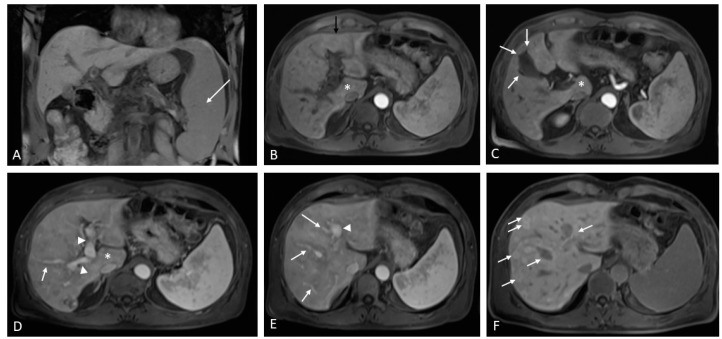
A patient with Crohn’s disease was found to have a low platelet count and splenomegaly. On MRI with hepatobiliary contrast, splenomegaly can be clearly observed along the longitudinal axis in the coronal plane ((**A**), white arrow). Note is also made of mild caudate lobe hypertrophy ((**B**–**D**) asterisk) and hypotrophy of segment IV ((**B**), black arrow), which is unusual against a smooth outline. The gallbladder is thickened with fibrotic spiculations ((**C**), white arrows). The heterogeneous signal intensity of the liver parenchyma is more pronounced around the portal tracts, where it appears hypointense in the portal venous phase. Note is made of an altered caliber of the main portal vein ((**D**,**E**), arrowheads)) with numerous narrowed distal portal branches surrounded by a hypointense signal ((**D**,**E**), white arrows). In the hepatobiliary phase, note is made of hyperintensity surrounding the portal tracts, which is in keeping with porto-sinusoidal vascular disorder ((**F**), white arrows).

**Figure 11:**
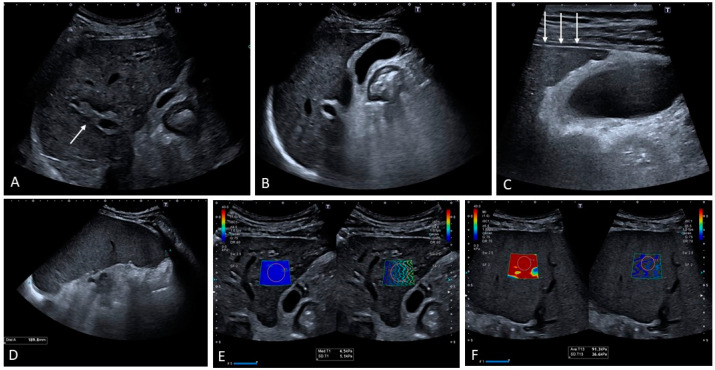
Patient with porto-sinusoidal vascular disorder (PSVD). Note is made of a heterogeneous echotexture with portal vein thickening surrounded by a hypoechoic halo ((**A**), white arrow). The gallbladder is thickened with a ‘spiculated’ outline in line with portal hypertension and fibrotic-related modifications (**B**,**C**). Note is made of a smooth liver outline against the heterogeneous echotexture ((**C**), arrows). Homogeneous splenomegaly is present; (**D**); liver stiffness is within normal range ((**E**), 4.5 kPa) but spleen stiffness is very high ((**F**), 91 kPa) in keeping with non-cirrhotic clinically significant portal hypertension.

**Figure 12:**
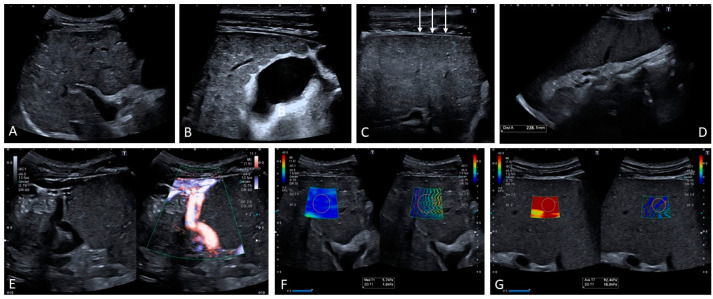
Another patient with porto-sinusoidal vascular disorder (PSVD). Note is made of a heterogeneous echotexture with portal vein thickening (**A**). The gallbladder is thickened with a ‘spiculated’ outline (**B**). Note is made of a smooth liver outline against the heterogeneous echotexture ((**C**) arrows). Homogeneous splenomegaly is present (**D**). Large splenorenal shunt (**E**). Liver stiffness is within normal range ((**F**), 5.7 kPa) while spleen stiffness is very high ((**G**), 92 kPa).

In some cases, PSVD can be misdiagnosed as chronic liver disease. However, there are some imaging features that, when taken altogether, suggest the diagnosis of PSVD with a higher accuracy. On ultrasound, the hepatic parenchyma is often heterogeneous, with periportal and peribiliary thickening which is sometimes surrounded by a hypoechoic halo that stands out against an ‘unusually’ smooth outline. This feature should raise the suspicion of PSVD as opposed to chronic liver disease, particularly in young patients with signs of PH and no other secondary cause. The gallbladder is also often thickened with a fibrotic and somewhat ‘spiculated’ appearance. Splenomegaly is a common finding, as well as the presence of portosystemic shunting. The natural history of PSVD can be characterized by the development of PVT and its assessment should be thoroughly checked in these patients with routine ultrasound follow-up. With regard to CT, a recent retrospective study comparing imaging of patients with PSVD and patients with cirrhosis revealed that reduced liver surface nodularity was highly predictive of PSVD with PH, compared to cirrhosis, and that accuracy was higher in the presence of normal or increased segment IV size, compared to cirrhosis. In addition, patients with advanced PSVD who showed increased surface nodularity and reduced segment IV size, which morphologically appear more similar to cirrhosis, had more advanced disease and poorer outcomes [19]. On MRI with hepatospecific contrast agents, liver surface nodularity was also found to be a distinctive feature to differentiate it from cirrhosis. In addition, both the presence of FNH-like lesions and especially periportal hyperintensity in the hepatobiliary phase are associated with PSVD (Figure 10) [20]. Finally, elastography has shown to be a very useful tool in this context. In fact, liver stiffness in PSVD is normal or slightly increased, while spleen stiffness reflects the severity of PH regardless of the underlying cause (Figure 11 and Figure 12) [21,22]. In conclusion, the predictive value of imaging findings for PSVD is particularly high, especially in the presence of signs of PH, heterogeneous echotexture, periportal thickening, smooth outline, and a high spleen/liver stiffness ratio. Nonetheless, to confirm a diagnosis of PSVD, liver biopsy is required, ideally by a transjugular approach, in order to also measure the hepatic venous pressure gradient (HVPG) during the same session. HVPG is usually relatively low and does not reflect the severity of PH owing to the presinusoidal component characteristic of this condition. However, it is important to highlight that HVPG can be high (>10 mmHg) in advanced cases, and only liver biopsy can exclude cirrhosis and provide a definitive diagnosis of PSVD [16].

## 4. Venous Outflow Obstruction Syndromes

This group of vascular liver diseases includes a variety of conditions that are characterized by an impairment of hepatic venous drainage, which may occur from the level of the sinusoids to the atrio-caval junction, or even include right heart chambers with consequent hepatic congestion and liver injury.

### 4.1. Budd–Chiari Syndrome

**Figure 13:**
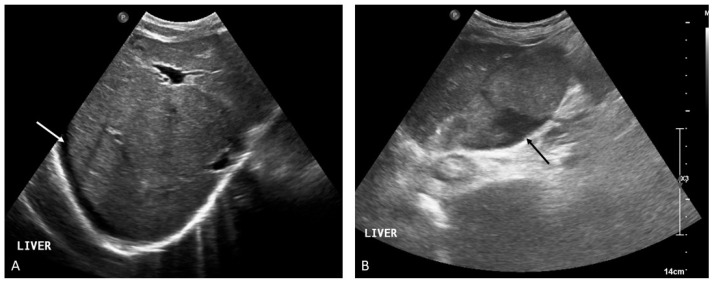
Subacute Budd–Chiari syndrome. The liver is enlarged and surrounded by a small amount of ascites ((**A**), white arrow). The hepatic veins are completely obliterated. The caudate lobe is grossly enlarged, with signs of ischemic infarction ((**B**), black arrow).

**Figure 14:**
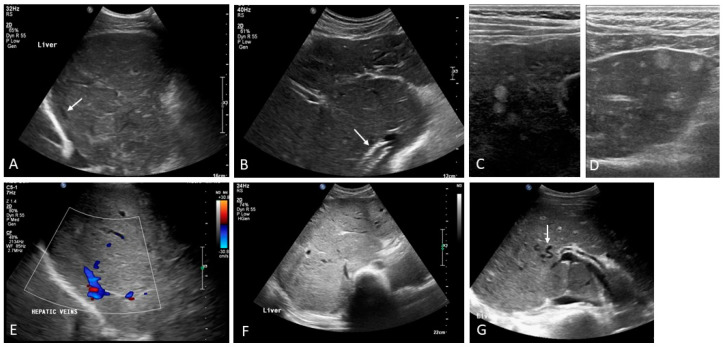
Patient with chronic Budd–Chiari. There is a remnant of the right hepatic vein while the other veins are not visible ((**A**), white arrow). Large caudate lobe hypertrophy and note is made of a transjugular intrahepatic portosystemic shunt (TIPS) in the inferior vena cava ((**B**), arrow). Multiple small rounded echogenic regenerative nodules are scattered throughout the parenchyma and better highlighted by a high-frequency transducer (**C**,**D**). Another case of Budd–Chiari syndrome (**E**–**G**). Note is made of small serpiginous intrahepatic veno-venous collaterals ((**G**), white arrow).

**Figure 15:**
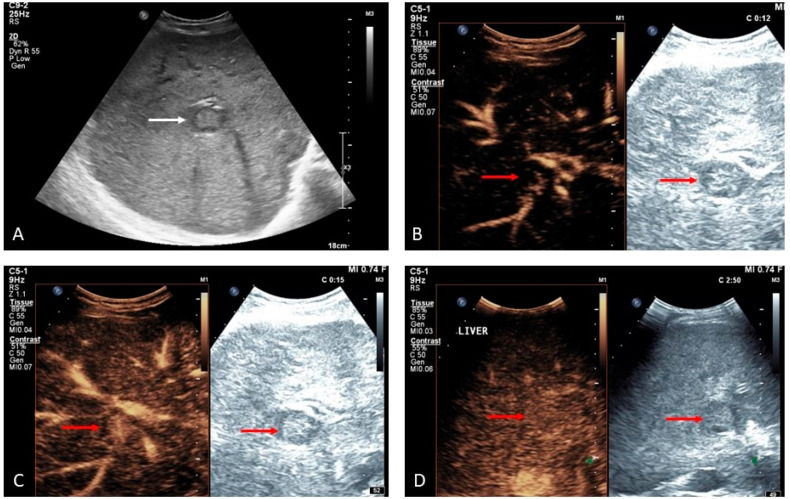
FNH-like lesion in a patient with Budd–Chiari syndrome ((**A**), white arrow). Note the centrifugal arterial enhancement ((**B**,**C**)), red arrows) and iso-enhancement in the late vascular phase ((**D**), red arrows).

**Figure 16:**
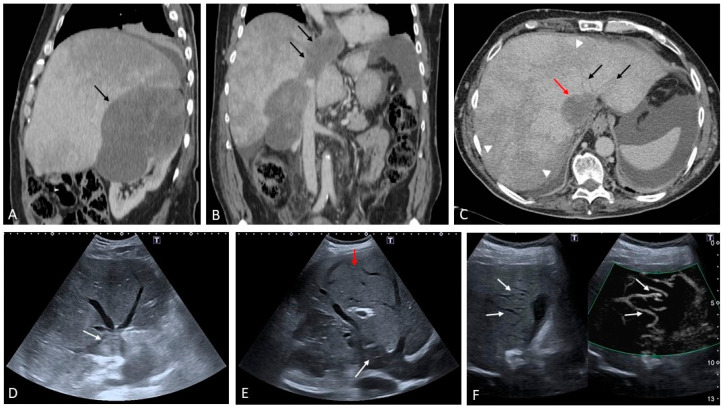
Secondary Budd–Chiari syndrome in a patient with a large adrenal carcinoma ((**A**), contrast-enhanced CT sagittal view, black arrow) complicated by neoplastic thrombosis invading the inferior vena cava with extension to the right atrium ((**B**), contrast-enhanced CT coronal view, black arrows). In (**C**), a transverse view shows the hypodense appearance of the thrombus in the IVC (red arrow) and congestion/blood stasis within the hepatic veins (black arrows). Note is made of the parenchymal heterogeneously perfused areas, typical of venous outflow obstruction (arrowheads). On B-mode US the large mass invading the IVC is easily detected in both transverse ((**D**), white arrow) and coronal views ((**E**), white arrow). Note is made of small serpiginous vascular channels between the distal segments of the hepatic veins ((**E**), red arrow) and between the hepatic veins and the venous drainage of the gallbladder ((**F**), white arrows).

**Figure 17:**
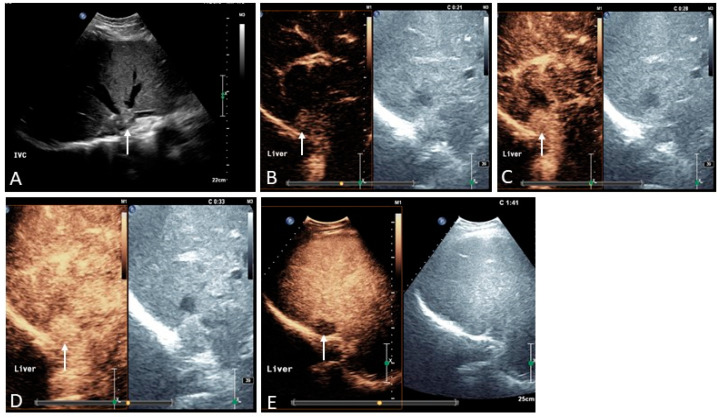
Budd–Chiari syndrome secondary to inferior vena cava thrombosis ((**A**), arrow). Contrast-enhanced ultrasound reveals enhancement of the thrombus in the arterial phase ((**B**–**D**), arrows) and subsequent washout in the following vascular phase ((**E**), arrow) in keeping with neoplastic invasion of the inferior vena cava.

Budd–Chiari syndrome (BCS) refers to hepatic venous obstruction at any level, from the small hepatic venules to the atrio-caval junction [5,23]. The outflow obstruction leads to sinusoidal congestion and ischemic liver injury that can lead to liver failure in cases of diffuse acute IVC and/or hepatic veins occlusion. Under other circumstances, it can have a more indolent course and manifest when fibrosis and related cirrhosis have already occurred. BCS is classified as primary and secondary. Primary causes are characterized by intraluminal thrombotic obstruction of the hepatic veins or IVC. The most frequent cause is thrombophilia secondary to congenital or acquired causes. In other circumstances, parasitic infections, cysts, trauma or neoplastic invasion of the hepatic veins or IVC can lead to hepatic venous outflow obstruction and hence BCS [24,25,26]. Another cause more frequently described in the Middle East or Asian countries is the presence of an inferior vena cava web that interferes with appropriate hepatic venous drainage. Caval webs are usually seen as a thin reticulin of fibrin within the lumen of the IVC localized between the atrio-caval junction and the site of drainage of the hepatic veins into the IVC [27]. In general, imaging findings are different, according to the onset of obstruction, and depend on the underlying cause and speed of development of the occlusion. Acute BCS manifests typically with abdominal pain and distension as well as signs of acute liver failure, since the liver does not have time to adapt to the lack of venous drainage. This condition represents a medical emergency because if the obstruction is not resolved, it can lead to severe ischemic hepatitis and liver failure. When the cause is secondary to a thrombotic occlusion, anticoagulation should be started immediately, but also an interventional procedure such as stenting and eventually transjugular intrahepatic portosystemic shunt (TIPS) should be considered according to the clinical scenario [28]. In the case of severe liver failure, poor response to anticoagulation and interventional attempts of recanalization or stenting, then liver transplantation should be considered [29,30]. Subacute-chronic Budd–Chiari syndrome often presents with abdominal discomfort, hepatomegaly and ascites. Imaging reveals hepatomegaly, obliteration of at least two hepatic veins and pronounced caudate lobe hypertrophy. Generally, in this phase, intrahepatic serpiginous veno-venous collaterals start developing and can be visualized easily on both ultrasound and cross-sectional imaging in an attempt to bypass the obliterated venous drainage. In CT or MRI, together with signs of hepatic vein or IVC obliteration, a typical ‘nutmeg’ appearance of liver parenchyma as an expression of diffuse congestion can also be observed. Occasionally, liver infarction can also be present, caudate lobe hypertrophy is typically very pronounced owing to its independent drainage into the IVC (Figure 13) [31,32,33]. In chronic BCS, the occluded hepatic veins are usually retracted and sometimes barely visible, or show fibrotic remnants. Serpiginous intrahepatic and subcapsular collaterals at this stage are more prominent. The caudate lobe is particularly hypertrophic. The liver outline is smooth or slightly irregular. Multiple regenerative nodules and FNH-like lesions are scattered throughout the hepatic parenchyma (Figure 14 and Figure 15), some of which can be quite large, reaching 4–5 cm in diameter [30,31,32]. Ultrasound offers excellent accuracy for diagnosing BCS, and contrast-enhanced CT is also very useful because it provides a panoramic view and allows the evaluation of vascular collateral circulation as a sign of severe PH that in BCS has a different distribution compared to other causes of liver cirrhosis. In BCS, the most commonly described collateral circulation systems refer to the left renal-hemiazygos pathway and the most frequently described vertebra lumbar-azygos pathway. Intrahepatic veno-venous collaterals and subcapsular collaterals are also a typical finding [30,34]. In addition, CECT is very accurate at evaluating liver involvement in secondary BCS (Figure 16). CEUS is useful in highlighting the presence of hepatic vein thrombosis, and similarly to PVT, it is very useful in the differential diagnosis between neoplastic invasion and bland thrombus (Figure 17).

### 4.2. Sinusoidal Obstruction Syndrome

Sinusoidal obstruction syndrome (SOS) is a condition characterized by hepatic sinusoidal obliteration secondary to endothelial damage caused by drug toxicity, radiotherapy, and allogenic or autologous hematopoietic stem cell transplantation (HSCT), which is the most commonly reported cause (14%) [5,34,35,36]. The occlusion of the sinusoids leads to hepatic congestion, ischemic liver damage, and eventually PH [37]. However, SOS can initially be completely asymptomatic and then subsequently present with jaundice, painful hepatomegaly, weight gain, and ascites. SOS can also present with multiorgan failure, with poor prognosis and a mortality rate of up to 80% [38]. The available Seattle and Baltimore Criteria rely on clinical and biochemical signs which, although non-specific, are considered an expression of SOS but are meant to be evaluated at 21 days post-HSCT, with confirmation often requiring liver biopsy, and ideally, HVPG measurement. More recently, an early diagnosis and prompt treatment with defibrotide has been shown to improve prognosis. Considering such a severe and potentially poor outcome, the identification of early predictors of SOS is critical [39].

**Figure 18:**
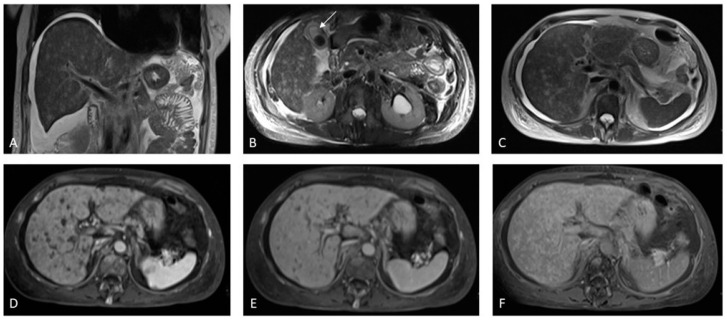
Patient with sinusoidal obstruction syndrome post-chemotherapy for breast cancer. The clinical onset was characterized by right upper quadrant pain, jaundice, and abdominal distension secondary to ascites. Blood tests revealed increased transaminase and bilirubin levels, low serum albumin. The MRI demonstrates a liver heterogeneous pattern on the T2W images (**A**–**C**) that becomes more pronounced in the arterial phase with multiple hypointense nodules that fade in the portal venous phase (**D**,**E**). Note is made of a more diffuse hypointense reticular pattern on the T1W post hepatocyte specific contrast injection (**F**). The latter is a feature which is highly specific for the diagnosis of sinusoidal obstruction syndrome. Note also the ascites (**A**–**C**) and thick-walled gallbladder ((**B**), white arrow).

SOS has a variable appearance in imaging depending on both the time of onset and the imaging modality. Initially, there may not be any detectable morphologic changes; however, in ultrasound with color Doppler, threaded hepatic veins and a reduced and pulsatile portal venous flow, followed by an increased arterial peak systolic velocity and resistive index, are some of the first hemodynamic consequences of SOS. Further reduction of the portal venous flow and its inversion, along with gallbladder wall thickening, are other described signs that increase in specificity depending on the clinical context. Liver parenchyma can have a heterogeneous appearance, and multiple small nodularities may be present along with a variable amount of ascites. By measuring liver stiffness, elastography can detect increased intrahepatic pressure as an expression of venous outflow obstruction. This is very important if we consider that hepatobiliary modifications related to SOS can initially show up as undetectable or subtle in imaging, thus understating the underlying pathological process. With regard to CECT, it may reveal periportal oedema, narrowed hepatic veins, multiple nodularities, thickened gallbladder wall, and ascites. The presence of a mosaic-like enhancement or nutmeg appearance as an expression of hepatic congestion—but with narrow hepatic veins—is suggestive of SOS, especially within a compatible clinical context. With regard to CEMRI, it demonstrates similar findings to CT. However, more recently, it has been shown that the presence of a diffuse hypointense reticular pattern on a post-contrast T1 delayed hepatobiliary phase is highly specific to SOS. Amongst the different imaging modalities, ultrasound with color Doppler and liver elastography is accurate, cost-effective, and easily reproducible, thus representing the modality of choice in patients who are due to undergo HSCT. In this selected population, patients should be assessed prior to HSCT to establish the normality of liver appearance and its stiffness values. Repeated evaluations post-HSCT during the following days/weeks allow for the prediction of disease development. Since the occurrence of SOS as a consequence of an adverse reaction to radiotherapy or chemotherapy is less common, there is currently no standardized protocol advised in these cases in contradistinction to HSCT. Hence, when SOS has usually reached a more advanced stage by the time it is diagnosed in non-HSCT patients. Ultrasound and elastography might be useful also in these cases, although CECT and especially CEMRI, in view of the known oncological history of the patient, are more accurate in facilitating the diagnosis (Figure 18) [40,41,42,43].

## 5. Hepatic Peliosis

‘Peliosis hepatis’ is a rare benign vascular condition characterized by the proliferation of liver sinusoids resulting in multiple blood-filled cystic lesions. The exact pathogenesis is still unknown, but different hypotheses have been proposed, including the following:

(i) altered sinusoidal flow and obstruction
(ii) hepatocellular necrosis
(iii) direct injury to the sinusoidal barrier [44].

According to the underlying histopathological characteristics, peliotic lesions are distinguished as ‘parenchyma type, in which sinusoidal endothelium is present, or ‘phlebectatic type’, in which the endothelium has been disrupted [45]. Hepatic peliosis (HP) can be found as a single lesion or multiple lesions of different sizes, ranging from a few millimeters to more than 4 cm [46]. Although the liver is the most common organ to be involved, it can also be found in the spleen, lymph nodes, bone marrow, ileum, and kidneys [47]. HP is labelled as idiopathic in 20–50% of cases, while in the majority of cases, it is described as being related to drugs such as corticosteroids, oral contraceptives, anabolic steroids, immunosuppressive therapy, and toxins. Chronic infectious diseases (such as AIDS), granulomatous infectious diseases (like tuberculosis), hematological diseases and inflammatory diseases have been found to be associated with HP [48]. Secondary HP seems to respond well to treatment of the underlying condition. Therefore, correct diagnosis is essential in order to initiate specific treatment. The appearance of HP in imaging may vary considerably; this is probably due to the histopathological structure, as well as the presence of bleeding within the lesion itself. Although imaging has an important role in diagnosis and follow-up, a biopsy of the lesion is almost always mandatory to confirm the diagnosis. This is due to the rarity of the condition and the sometimes-overlapping pattern of enhancement with malignancies (contrast arterial enhancement and portal and delayed vascular phase washout) [46]. In CECT, HP can show different patterns of enhancement. It can be hypoattenuating and become progressively iso-attenuating to the hepatic parenchyma. In some instances, they can display an early peripheral hyperenhancement and accumulation of contrast within the lesion known as the ‘target sign’. Ultrasound findings are quite heterogenous and do not have a specific sonographic appearance. HP lesions are typically homogeneous and hypoechoic on a background of a steatotic liver; hyperechoic on the background of a normal echogenic liver; or, alternatively, a mixed pattern of echogenicities and heterogeneity when complicated by bleeding. Usually, these lesions do not exert mass effect, and this appears to be a distinctive feature [48]. On CEUS, the most frequent pattern is of a heterogeneous hyperenhancement in the arterial phase, with washout in the portal and late vascular phases that initially suggests malignancy, thus requiring histological confirmation for a definitive diagnosis [48]. Peripheral ring enhancement with centripetal filling and persistent hyperenhancement in the late vascular phase have been described. In some lesions, a transient ‘fast surge’ echo-enhancement is found centrally with no centripetal enhancement [49]. The appearance of hepatic peliosis on MR examination depends mainly on the status of the blood component. On T2-weighted sequences, peliotic lesions are usually hyperintense to liver parenchyma, with multiple foci of high signal, likely attributable to hemorrhagic necrosis. On T1-weighted images after contrast material injection, peliotic lesions usually show a centrifugal enhancement. However, a recent report described an unusual centripetal enhancement pattern which may be confused with that of a hemangioma [50,51].

## 6. Vascular Malformations

**Figure 19:**
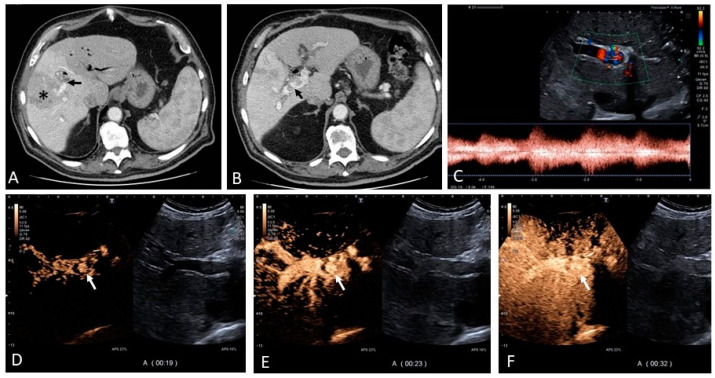
Contrast-enhanced CT shows a large right lobe hepatocellular carcinoma ((**A**), asterisk) with portal vein invasion ((**A**), black arrow) and an arterio-portal fistula ((**B**), black arrow). Ultrasound color Doppler shows intra-portal aliasing with turbulent arterial high peak systolic velocities as well as high diastolic velocities in keeping with an arterio-portal fistula (**C**). Contrast-enhanced ultrasound highlights the site of the fistula (white arrows) and early arterial enhancement of the portal vein as a result of the shunt (**D**–**F**).

**Figure 20:**
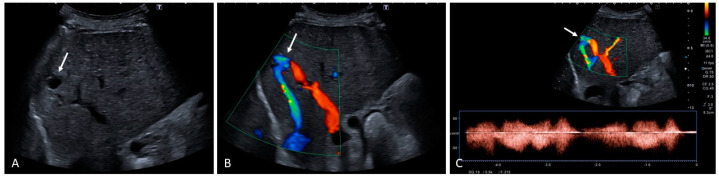
There is a small round anechoic area that resembles a simple cyst in segment VII ((**A**), white arrow). The use of color Doppler reveals that the rounded anechoic area is vascular and actually the point of aberrant connection between the right hepatic vein and the right portal vein branches ((**B**), white arrow). The Doppler signal highlights the turbulence of the mixed flow at the site of the vascular aberrant communication ((**C**), white arrow).

When describing vascular malformations of the liver (VMs), these aberrant communications should be distinguished from those occurring in cirrhosis, where their development is driven by PH. In contrast, nosological vascular diseases are either congenital or acquired, eventually causing PH rather than being caused by it. Among the acquired VMs, arterio-portal fistulae (APF), and arterio-venous fistulae (AVF) can be a consequence of hepatocellular carcinoma (especially when the portal vein is invaded) (Figure 19), trauma and interventional procedures (liver biopsies, percutaneous biliary drainages, and surgery) [52]. They are usually diagnosed via imaging performed for other reasons or when undergoing investigations to identify causes of abnormal liver function tests or unknown causes of PH. On ultrasound, the presence of intrahepatic anechoic rounded or convoluted tubular structures refers to the aberrant vascular channels. Usually, the exact site of communication between two different vessels is characterized through color Doppler by the presence of a mixed color-pattern termed the ‘ying-yang’ sign or pronounced color aliasing that reflects blood flow turbulence with high velocities at the level of the site of vascular communication (Figure 20). In cases of arterio-portal fistulae or arterio-venous fistulae, an arterial Doppler signal is depicted respectively along the portal venous tract (Figure 19) or the hepatic venous tract that communicates with the fistula. On CT scan, the portal vein or hepatic vein typically shows enhancement in the arterial phase, which is a distinctive feature of the portal or hepatic vein that directly communicates with the hepatic artery (Figure 20). Splenic arterio-venous fistulas are a unique cause of non-cirrhotic pre-hepatic PH of vascular origin; they can be congenital or acquired and must be accounted for when investigating unknown causes of PH. Diagnostic imaging benefits from color Doppler analysis, and more importantly, CECT and angiography; treatment usually involves trans-arterial embolization [53,54].

**Figure 21:**
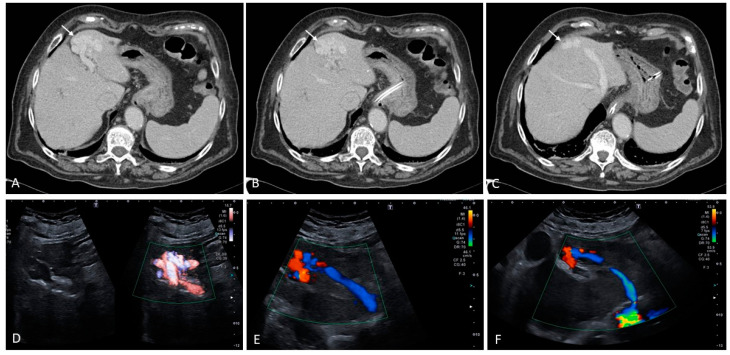
A 70-year-old was found clinically encephalopathic, with high levels of ammonia. No signs of chronic liver disease, but convoluted serpiginous vascular channels at the point of confluence between the left portal venous branch and the left hepatic vein are evident (arrows). Findings are compatible with a congenital intrahepatic portal systemic shunt between the left branch of the portal vein and the left hepatic vein. Contrast enhanced CT shows the portal-venous shunt from its more proximal to its distal venous portion ((**A**–**C**), white arrows). Color Doppler was useful to corroborate these findings (**D**–**F**) and follow-up until embolization was achieved.

Hepatic artery aneurysms are fusiform or saccular dilatations that can develop along different tracts of the hepatic arterial supply due to artherosclerosis (more often occurring extrahepatically), vasculitis, connective tissue disorders, and infectious or inflammatory causes [55]. Congenital portosystemic shunt(s) (CPSS) are rare vascular malformations originating from incomplete vascular remodeling between the embryonic and fetal hepatic and peri-hepatic circulation [56], through which intestinal blood flow bypasses the liver and reaches the systemic circulation unfiltered [57]. The shunt can occur completely outside the liver, known as extrahepatic CPSS type I (Abernethy malformation), or as type II malformations, in which some of the portal flow is maintained via a smaller anastomosis between the portal and systemic venous systems [58]. Related clinical findings include portosystemic encephalopathy, porto-pulmonary hypertension, and endocrine dysfunction [57]. Congenital intrahepatic CPSS occur inside the liver when the failed fusion of the right vitelline and umbilical venous plexus creates communication between the intrahepatic portal and hepatic or peri-hepatic veins (Figure 21) [59].

CPSS are typically associated with the development of liver lesions, most of which are FNHs or adenomas, although hepatocellular carcinomas have also been described in teenagers and adults [57,60,61]. Liver nodules in extrahepatic CPSS tend to increase in size more rapidly and have a higher risk of malignant transformation, probably owing to the absence/significant reduction of portal flow and increase of arterial supply. However, in intrahepatic CPSS, the lesions are usually benign and smaller in size [57]. Imaging plays a crucial role in the management of patients with CPSS, allowing for the assessment of the aberrant anatomy, the topography of the shunt, and planning of intervention, as well as the characterization of any focal liver lesions. Color Doppler analysis is very useful for evaluating the direction and flow velocities of the shunt, but CECT with a careful evaluation of all vascular phases, is crucial in providing a panoramic view of the shunt and its communications. MRI with hepatospecific contrast remains the imaging modality of choice for characterizing the liver masses that are associated with CPSS [58].

## 7. Hereditary Hemorrhagic Telangiectasia (Osler–Weber–Rendu Disease)

Hereditary hemorrhagic telangiectasia (HHT) is an autosomal dominant hereditary disease characterized by diffuse cutaneous, mucosal, visceral, and cerebral vascular malformations [62,63]. Telangiectasias are derived from dilated post-capillary venules which enlarge and fuse with arterioles, bypassing the capillary system and thus leading to arterio-venous communications. HHT is often underdiagnosed and can occasionally be depicted in imaging, or as a consequence of investigations secondary to gastrointestinal bleeding [64]. Visceral VMs can involve the brain, liver, and lung.

The liver is the most common site of VMs in patients with HHT, being described in 41–84% of patients [65,66]. Hepatic HHT can evolve from small telangiectasias into large VMs. Given the dual blood supply to the liver, three different types of shunting, which can occur concomitantly, are possible:

1. Arterio-venous (hepatic artery to hepatic vein)
2. Arterio-portal (hepatic artery to portal vein)
3. Porto-venous (portal vein to hepatic vein).

Liver involvement is often asymptomatic but can also lead to different and possibly coexisting clinical features: high-output cardiac failure, PH, hepatic encephalopathy, and biliary/mesenteric ischemia. These perfusion abnormalities can also entail hepatocellular regenerative activity, leading to the development of FNHs or to nodular regenerative hyperplasia (NRH) [67]. Although CECT scans offer a panoramic evaluation and may provide details on the vascular connection, ultrasound has a fundamental role in diagnosis, staging and follow-up. The following early signs of liver involvement in HHT are detectable by US:

1. Hepatic artery dilatation > 4 mm.
2. Normally, the diameter of the hepatic artery is smaller than that of the splenic artery; thus, an inversion of this relationship may be an early sign of liver VM.
3. An increased velocity within the hepatic artery, with a peak flow velocity greater than 100 cm/s.
4. Peripheral subcapsular ‘spots’ on power or color Doppler with high arterial blood flow velocities and low resistive indices are suggestive of small peripheral VMs.
5. Other ultrasound findings include tortuous intrahepatic tubular structures communicating between the portal venous branches and the hepatic venous branches, tubular structures parallel to the portal branches representing dilated arterial branches, and vascular shunts. Arterio-systemic and arterio-portal shunts have low resistive indices, and arterio-portal shunts may be accompanied by the presence of portosystemic collaterals. Furthermore, arterio-systemic shunts can lead to arterialization, dilation, and turbulent flow of the portal vein and hepatic veins.

In advanced stages of HHT, the alterations of vascular flow lead to structural changes, and eventually fibrosis with an appearance that resembles cirrhosis, although the hepatocellular architecture is still preserved. For this reason, it is termed ‘pseudocirrhosis’. In 2003, Caselitz et al. proposed a ultrasound criteria for hepatic involvement in HHT [68]. The classification of Buscarini et al. [69] highlights the usefulness of Color Doppler analysis in the classification and evaluation of the severity of HHT. Buscarini proposed 5 grades of severity—0+, 1, 2, 3, and 4—along with the following criteria:

Grade 0+: The signs are very difficult to detect with ultrasound, but it would be useful to identify some in order to keep them under review over time. The characteristics of grade 0+ are a HA diameter between 5 and 6 mm, and/or a peak flow velocity (PFV) >80 cm/s, and/or -a resistive index (RI) <0.55, and/or peripheral hepatic hypervascularization.

Grade 1: HA dilatation, only extrahepatic > 6 mm, and PFV > 80 cm/s, and/or RI < 0.55.

**Figure 22:**
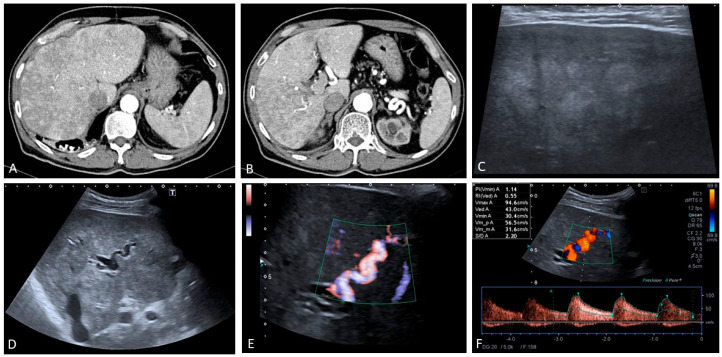
Patient with hereditary haemorrhagic telangiectasia and liver involvement. Note is made of a large area of diffuse heterogeneous enhancement on the arterial phase of this contrast-enhanced CT (**A**,**B**). There is also an irregular outline that resembles chronic liver disease (‘pseudocirrhotic pattern’) (**A**). On ultrasound, a heterogeneous echotexture is present with a patchy echogenic pattern and pseudonodularities in keeping with heterogeneous perfusional areas owing to the marked arterialized parenchyma (**C**,**D**). Pronounced arterial hypertrophy can also be noted with a typical double channel appearance (**E**) and high peak systolic velocities > 80 cm/s (**F**).

Grade 2: HA dilatation, extra- and intrahepatic ‘double channel’ and a PFV > 80 cm/s, possibly associated with moderate flow abnormality the hepatic and/or portal veins (Figure 22).

Grade 3: Complex changes in the hepatic artery and its branches (tortuous and tangled) with marked flow abnormalities associated with moderate dilatation of the hepatic and/or portal veins, and/or abnormality of the hepatic and/or portal vein flow.

Grade 4: Decompensation of arterio-venous shunts with marked dilatation of the hepatic and/or portal vein(s), as well as marked flow abnormalities in both the arteries and vein(s).

Grades 0+, 1 and 2 are completely asymptomatic. In grades 3 and 4, it is possible to find an increase in cholestasis markers, a finding that requires caution in view of the potential risk of necrotizing cholangitis [70]. According to Buscarini, for grades 1 and 2, interval Doppler US is sufficient for follow-up; however, grades 3 and 4 with cardiac overload at follow-up require echocardiography.

## 8. Summary

Complementary imaging modalities play an important role in the diagnosis of VLDs. Both CECT and CEMRI are excellent methods to assess the liver parenchyma, tissue perfusion as well as the complex vascular interconnections that characterise some of these conditions. While CECT is the modality of choice to evaluate splanchnic circulation and intra- and extrahepatic collateral vascular pathways, CEMRI with hepatobiliary contrast is considered the gold standard to differentiate focal lesions and highlight parenchymal and periportal abnormalities that are of high diagnostic value, as seen more specifically in SOS and PSVD.

Multiparametric ultrasound also has an extremely important role in the diagnosis of VLDs, delivering an in-depth evaluation of liver appearance thanks to the high-frequency transducers, which can highlight details on the surface of the liver and parenchymal abnormalities that otherwise could be overlooked. The use of colour Doppler is very important, as it can be used to acquire real-time information on flow direction and velocities and reveal the presence of shunts and vascular fistulae. In some conditions, such as SOS and HHT, B-mode ultrasound and colour Doppler signs are used to create diagnostic and staging scoring systems that are highly predictive of both diagnosis and prognosis. Sulfur hexafluoride-based ultrasound contrast agents are pure blood pool agents that highlight different liver enhancement patterns according to its circulation (arterial, portal, and late venous phase), with very high accuracy in distinguishing bland thrombus from neoplastic vascular invasion.

Finally, the use of elastography in this clinical context goes beyond the evaluation of liver fibrosis and can be applied to rapidly distinguish cirrhotic from non-cirrhotic portal hypertension by evaluating both liver and spleen stiffness and their ratio. Liver elastography also has a clear role in predicting the occurrence of SOS in patients undergoing HSCT.

## 9. Conclusions

The three main cross-sectional (CT/MRI/Ultrasound) imaging modalities provide crucial information from different perspectives, enabling both radiologists and hepatologists to deal with the diagnosis and management of these unique vascular disorders. Notably, ultrasound distinguishes itself by being able to provide real-time information on the appearance of the liver anatomy and texture, as well as provide a handle on the hepatic circulation and biomechanical properties of the hepatosplenic axis with high accuracy, repeatability and radiation-free benefits. It is therefore the modality of choice not only for highlighting findings that aid further diagnostic investigations, but also for a point-of-care follow-up approach once second-line imaging is used to integrate and complete the diagnostic workup of VLDs.

## References

[1] Boccatonda A., Gentilini S., Zanata E., Simion C., Serra C., Simioni P., Piscaglia F., Campello E., Ageno W. (2024). Portal VeinThrombosis: State-of-the-Art Review. J. Clin. Med..

[2] Monaco G., Bucherini L., Stefanini B., Piscaglia F., Foschi F.G., Ielasi L. (2023). Direct oral anticoagulants for the treatment of splanchnic vein thrombosis: A state of art. World J. Gastroenterol..

[3] Trebicka J., Strassburg C.P. (2014). Etiology and Complications of Portal Vein Thrombosis. Viszeralmedizin.

[4] Ali K. (2020). Abu-Alfa, Use of Gadolinium-Based Contrast Agents in Kidney Disease Patients: Time for Change. Am. J. Kidney Dis..

[5] Garcia-Pagàn J.C., Buscarini E., Janssen H.L., Leebeck F.W.G., Plessier A., Rubbia-Brandt L., Senzolo M., Schouten J.N.L., Tripodi A., Valla D.C. (2016). EASL clinical practice guidelines: Vascular diseases of the liver. J. Hepatol..

[6] Chen J., Zhu J., Zhang C., Song Y., Huang P. (2020). Contrast-enhanced ultrasound for the characterization of portal vein thrombosis vs tumor-in-vein in HCC patients: A systematic review and meta-analysis. Eur. Radiol..

[7] Khuroo M.S., Rather A.A., Khuroo N.S., Khuroo M.S. (2016). Portal biliopathy. World J. Gastroenterol..

[8] Fusaro L., Di Bella S., Martingano P., Crocè L.S., Giuffrè M. (2023). Pylephlebitis: A Systematic Review on Etiology, Diagnosis, and Treatment of Infective Portal Vein Thrombosis. Diagnostics.

[9] Le Roy B., Gelli M., Serji B., Memeo R., Vibert E. (2015). Portal biliopathy as a complication of extrahepatic portal hypertension: Etiology, presentation and management. J. Visc. Surg..

[10] Tarantino L., Francica G., Sordelli I., Esposito F., Giorgio A., Sorrentino P., de Stefano G., Di Sarno A., Ferraioli G., Sperlongano P. (2006). Diagnosis of benign and malignant portal vein thrombosis in cirrhotic patients with hepatocellular carcinoma: Color Doppler US, contrast-enhanced US, and fine-needle biopsy. Abdom. Imaging.

[11] Tarantino L., Ambrosino P., Di Minno M.N. (2015). Contrast-enhanced ultrasound in differentiating malignant from benign portal vein thrombosis in hepatocellular carcinoma. World J. Gastroenterol..

[12] Cerrito L., Ainora M.E., Di Francesco S., Galasso L., Gasbarrini A., Zocco M.A. (2023). The Role of Contrast-Enhanced Ultrasound (CEUS) in the Detection of Neoplastic Portal Vein Thrombosis in Patients with Hepatocellular Carcinoma. Tomography.

[13] Anton A., Campreciós G., Pérez-Campuzano V., Orts L., García-Pagán J.C., Hernández-Gea V. (2022). The Pathophysiology of Portal Vein Thrombosis in Cirrhosis: Getting Deeper into Virchow’s Triad. J. Clin. Med..

[14] Stine J.G., Wang J., Shah P.M., Argo C.K., Intagliata N., Uflacker A., Caldwell S.H., Northup P.G. (2018). Decreased portal vein velocity is predictive of the development of portal vein thrombosis: A matched case-control study. Liver Int..

[15] European Association for the Study of the Liver (2022). EASL Clinical Practice Guidelines on prevention and management of bleeding and thrombosis in patients with cirrhosis. J. Hepatol..

[16] Tublin M.E., Towbin A.J., Federle M.P., Nalesnik M.A. (2008). Altered liver morphology after portal vein thrombosis: Not always cirrhosis. Dig. Dis. Sci..

[17] De Gottardi A., Sempoux C., Berzigotti A. (2022). Porto-sinusoidal vascular disorder. J. Hepatol..

[18] De Gottardi A., Rautou P.E., Schouten J., Rubbia-Brandt L., Leebeek F., Trebicka J., Murad S.D., Vilgrain V., Hernandez-Gea V., Nery F. (2019). Porto-sinusoidal vascular disease: Proposal and description of a novel entity. Lancet Gastroenterol. Hepatol..

[19] Valainathan S.R., Sartoris R., Elkrief L., Magaz M., Betancourt F., Pellegrino S., Nivolli A., Dioguardi Burgio M., Flattet Y., Terraz S. (2022). Contrast-enhanced CT and liver surface nodularity for the diagnosis of porto-sinusoidal vascular disorder: A case-control study. Hepatology.

[20] Lampichler K., Semmler G., Wöran K., Simbrunner B., Jachs M., Hartl L., Bauer D.J.M., Balcar L., Burghart L., Trauner M. (2023). Imaging features facilitate diagnosis of porto-sinusoidal vascular disorder. Eur. Radiol..

[21] Ferreira-Silva J., Gaspar R., Liberal R., Cardoso H., Macedo G. (2023). Splenic-hepatic elastography index is useful in differentiating between porto-sinusoidal vascular disease and cirrhosis in patients with portal hypertension. Dig. Liver Dis..

[22] Elkrief L., Lazareth M., Chevret S., Paradis V., Magaz M., Blaise L., Rubbia-Brandt L., Moga L., Durand F., Payancé A. (2021). ANRS CO12 CirVir Group. Liver Stiffness by Transient Elastography to Detect Porto-Sinusoidal Vascular Liver Disease with Portal Hypertension. Hepatology.

[23] Valla D.C. (2018). Budd-Chiari syndrome/hepatic venous outflow tract obstruction. Hepatol. Int..

[24] Aydinli M., Bayraktar Y. (2007). Budd-Chiari syndrome: Aetiology, pathogenesis and diagnosis. World J. Gastroenterol..

[25] Ludwig J., Hashimoto E., McGill D.B., van Heerden J.A. (1990). Classification of hepatic venous outflow obstruction: Ambiguous terminology of the Budd-Chiari syndrome. Mayo Clin. Proc..

[26] Senzolo M., Cholongitas E.C., Patch D., Burroughs A.K. (2005). Update on the classification, assessment of prognosis and therapy of Budd-Chiari syndrome. Nat. Clin. Pract. Gastroenterol. Hepatol..

[27] Bozorgmanesh A., Selvam D.A., Caridi J.G. (2007). Budd-Chiari syndrome: Hepatic venous web outflow obstruction treated by percutaneous placement of hepatic vein stent. Semin. Interv. Radiol..

[28] Lombardo S., Espejo J.J., Pérez-Montilla M.E., Zurera L.J., González-Galilea Á. (2018). The keys to successful TIPS in patients with portal vein thrombosis and cavernous transformation. Radiologia (Engl. Ed.).

[29] Magaz M., Soy G., García-Pagán J.C. (2020). Budd-Chiari Syndrome: Anticoagulation, TIPS, or Transplant. Curr. Hepatol. Rep..

[30] Brancatelli G., Vilgrain V., Federle M.P., Hakime A., Lagalla R., Iannaccone R., Valla D. (2007). Budd-Chiari Syndrome; Spectrum of Imaging Findings. Am. J. Roentgeneology.

[31] Porrello G., Mamone G., Miraglia R. (2023). Budd-Chiari Syndrome Imaging Diagnosis: State of the Art and Future Perspectives. Diagnostics.

[32] Bansal V., Gupta P., Sinha S., Dhaka N., Kalra N., Vijayvergiya R., Dutta U., Kochhar R. (2018). Budd-Chiari syndrome: Imaging review. Br. J. Radiol..

[33] Noone T.C., Semelka R.C., Siegelman E.S., Balci N.C., Hussain S.M., Kim P.N., Mitchell D.G. (2000). Budd-Chiari syndrome: Spectrum of appearances of acute, subacute, and chronic disease with magnetic resonance imaging. J. Magn. Reson. Imaging.

[34] Cho O.K., Koo J.H., Kim Y.S., Rhim H.C., Koh B.H., Seo H.S. (1996). Collateral Pathways in Budd-Chiari Syndrome: CT and Venographic Correlation. AJR.

[35] Carreras E., Díaz-Beyá M., Rosiñol L., Martínez C., Fernández-Avilés F., Rovira M. (2011). The incidence of veno-occlusive disease following allogeneic hematopoietic stem cell transplantation has diminished and the outcome improved over the last decade. Biol. Blood Marrow Transplant..

[36] Mohty M., Malard F., Abecassis M., Aerts E., Alaskar A.S., Aljurf M., Arat M., Bader P., Baron F., Bazarbachi A. (2015). Sinusoidal obstruction syndrome/veno-occlusive disease: Current situation and perspectives-a position statement from the European society for blood and marrow transplantation (EBMT). Bone Marrow Transpl..

[37] DeLeve L.D., Shulman H.M., McDonald G.B. (2002). Toxic injury to hepatic sinusoids: Sinusoidal obstruction syndrome (veno-occlusive disease). Semin. Liver Dis..

[38] Coppell J.A., Richardson P.G., Soiffer R., Martin P.L., Kernan N.A., Chen A., Guinan E., Vogelsang G., Krishnan A., Giralt S. (2010). Hepatic veno-occlusive disease following stem cell transplantation: Incidence, clinicalcourse, and outcome. Biol. Blood Marrow Transplant..

[39] Richardson P.G., Smith A.R., Triplett B.M., Kernan N.A., Grupp S.A., Antin J.H., Lehmann L., Miloslavsky M., Hume R., Hannah A.L. (2017). Earlier defibrotide initiation post-diagnosis of veno-occlusive disease/sinusoidal obstruction syndrome improves Day +100 survival following haematopoietic stem cell transplantation. Br. J. Haematol..

[40] Bohte A.E., Dierselhuis M.P., van Noesel M.M., Lequin M.H. (2022). Imaging features of hepatic sinusoidal obstruction syndrome or veno-occlusive disease in children. Pediatr. Radiol..

[41] Zhang Y., Yan Y., Song B. (2019). Noninvasive imaging diagnosis of sinusoidal obstruction syndrome: A pictorial review. Insights Imaging.

[42] Chan S.S., Colecchia A., Duarte R.F., Bonifazi F., Ravaioli F., Bourhis J.H. (2020). Imaging in Hepatic Veno-Occlusive Disease/Sinusoidal Obstruction Syndrome. Biol. Blood Marrow Transplant..

[43] Ravaioli F., Colecchia A., Alemanni L.V., Vestito A., Dajti E., Marasco G., Sessa M., Pession A., Bonifazi F., Festi D. (2019). Role of imaging techniques in liver veno-occlusive disease diagnosis: Recent advances and literature review. Expert Rev. Gastroenterol. Hepatol..

[44] Wanless I.R., Huang W.Y. (2012). Vascular disorders. MacSween’s Pathology of the Liver.

[45] Yanoff M., Rawson A.J. (1964). Peliosis hepatis. An anatomic study with demonstration of two varieties. Arch. Pathol..

[46] Iwata T., Adachi K., Takahashi M. (2017). Peliosis Hepatis Mimicking Malignant HypervascularTumors. J. Gastrointest. Surg..

[47] Crocetti D., Palmieri A., Pedullà G., Pasta V., D’Orazi V., Grazi G.L. (2015). Peliosis hepatis: Personal experience and literature review. World J. Gastroenterol..

[48] Dong Y., Wang W.P., Lim A.K., Lee W.J., Clevert D.A., Höpfner M., Tannapfel A., Dietrich C.F. (2021). Ultrasound findings in peliosis hepatis. Ultrasonography.

[49] Kleinig P., Davies R.P., Maddern G., Kew J. (2003). Peliosis hepatis: Central “fast surge” ultrasound enhancement and multislice CT appearances. Clin. Radiol..

[50] Ferrozzi F., Tognini G., Zuccoli G., Cademartiri F., Pavone P. (2001). Peliosis hepatis with pseudotumoral and hemorrhagic evolution: CT and MR findings. Abdom. Imaging.

[51] Gouya H., Vignaux O., Legmann P., de Pigneux G., Bonnin A. (2001). Peliosis hepatis: Triphasic helical CT and dynamic MRI findings. Abdom. Imaging.

[52] Cao B., Tian K., Zhou H., Li C., Liu D., Tan Y. (2022). Hepatic Arterioportal Fistulas: A Retrospective Analysis of 97 Cases. J. Clin. Transl. Hepatol..

[53] Yassine A.A., Al Moussawi H., Kreidieh M., Dahabra L., Al-Roubaie M., Satapathy S. (2023). Splenic arteriovenous fistula leading to non-cirrhotic portal hypertension: A case report. Gastroenterol. Rep..

[54] Alexander E., Santos E. (2023). Endovascular management of incidentally discovered splenic arteriovenous fistula resulting from ruptured splenic aneurysm: Case report and review of the literature. Radiol. Case Rep..

[55] Haghighatkhah H., Sanei Taheri M., Kharazi S.M., Zamini M., Rabani Khorasgani S., Jahangiri Zarkani Z. (2019). Hepatic Artery Aneurysms as a Rare but Important Cause of Abdominal Pain; a Case Series. Arch. Acad. Emerg. Med..

[56] McLin V.A., Abella S.F., Debray D., Guérin F., Beghetti M., Savale L., Wildhaber B.E., Gonzales E. (2019). Congenital portosystemic shunts: Current diagnosis and management. J. Pediatr. Gastroenterol. Nutr..

[57] McLin V.A., Franchi-Abella S., Brütsch T., Bahadori A., Casotti V., de Ville de Goyet J., Dumery G., Gonzales E., Guérin F., Hascoet S. (2023). Expert management of congenital portosystemic shunts and their complications. JHEP Rep..

[58] Schmalz M.J., Radhakrishnan K. (2020). Vascular anomalies associated with hepatic shunting. World J. Gastroenterol..

[59] Papamichail M., Pizanias M., Heaton N. (2018). Congenital portosystemic venous shunt. Eur. J. Pediatr..

[60] Baiges A., Turon F., Simón-Talero M., Tasayco S., Bueno J., Zekrini K., Plessier A., Franchi-Abella S., Guerin F., Mukund A. (2020). Congenital extrahepatic portosystemic shunts (Abernethy malformation): An international observational Study. Hepatology.

[61] Sanada Y., Mizuta K., Niki T., Tashiro M., Hirata Y., Okada N., Yamada N., Ihara Y., Urahashi T., Soejima Y. (2015). Hepatocellular nodules resulting from congenital extrahepatic portosystemic shunts can differentiate into potentially malignant hepatocellular adenomas. J. Hepatobiliary Pancreat. Sci..

[62] Faughnan M.E., Mager J.J., Hetts S.W., Palda V.A., Lang-Robertson K., Buscarini E., Deslandres E., Kasthuri R.S., Lausman A., Poetker D. (2020). Second International Guidelines for the Diagnosis and Management of Hereditary Hemorrhagic Telangiectasia. Ann. Intern. Med..

[63] McDonald J., Wooderchak-Donahue W., VanSant Webb C., Whitehead K., Stevenson D.A., Bayrak-Toydemir P. (2015). Hereditary hemorrhagic telangiectasia: Genetics and molecular diagnostics in a new era. Front. Genet..

[64] Shovlin C.L., Guttmacher A.E., Buscarini E., Faughnan M.E., Hyland R.H., Westermann C.J., Kjeldsen A.D., Plauchu H. (2000). Diagnostic criteria for hereditary hemorrhagic telangiectasia (Rendu-Osler-Weber syndrome). Am. J. Med. Genet..

[65] Khalid S.K., Garcia-Tsao G. (2008). Hepatic vascular malformations in hereditary hemorrhagic telangiectasia. Semin. Liver Dis..

[66] Buonamico P., Suppressa P., Lenato G.M., Pasculli G., D’Ovidio F., Memeo M., Scardapane A., Sabbà C. (2008). Liver involvement in a large cohort of patients with hereditary hemorrhagic telangiectasia: Echo-color-Doppler vs multislice computed tomography study. J. Hepatol..

[67] Buscarini E., Danesino C., Plauchu H., de Fazio C., Olivieri C., Brambilla G., Menozzi F., Reduzzi L., Blotta P., Gazzaniga P. (2004). High prevalence of hepatic focal nodular hyperplasia in subjects with hereditary hemorrhagic telangiectasia. Ultrasound Med. Biol..

[68] Caselitz M., Bahr M.J., Bleck J.S., Chavan A., Manns M.P., Wagner S., Gebel M. (2003). Sonographic criteria for the diagnosis of hepatic involvement in hereditary hemorrhagic telangiectasia (HHT). Hepatology.

[69] Buscarini E., Danesino C., Olivieri C., Lupinacci G., De Grazia F., Reduzzi L., Blotta P., Gazzaniga P., Pagella F., Grosso M. (2004). Doppler ultrasonographic grading of hepatic vascular malformations in hereditary hemorrhagic telangiectasia-results of extensive screening. Ultraschall Med..

[70] Kelly C., Buscarini E., Manfredi G., Gregory S., Heneghan M.A. (2024). Hepatic manifestations of hereditary haemorrhagic telangiectasia. Liver Int..

